# Counter Prescribing

**Published:** 1878-08

**Authors:** 


### Counter Prescribing.

From all accounts it would appear that the practice of counter-prescribing by druggists is on the increase, especially in the southern and eastern part of the city. Would it not be well, in the coming fall, for a committee of the County Society to investigate the matter and suggest a remedy? If these counter-leeches still persist in defying ordinary decency, they may only hasten the time when physicians will keep their own medicines.—*Med. Record.*

